# An Atypical Case of Toxic Epidermal Necrolysis Associated With Ceftriaxone

**DOI:** 10.7759/cureus.93640

**Published:** 2025-10-01

**Authors:** Krunal Shukla, Michael Barry, Eric J Basile, Jeffrey Budd

**Affiliations:** 1 Internal Medicine, University of Florida College of Medicine, Gainesville, USA; 2 Cardiovascular Disease, University of South Florida Morsani College of Medicine, Tampa, USA; 3 Internal Medicine, Malcom Randall Veterans Affairs Medical Center, Gainesville, USA

**Keywords:** ceftriaxone, ceftriaxone adverse effects, sjs, stevens-johnson syndrome and toxic epidermal necrolysis (sjs/ten), ten

## Abstract

Toxic epidermal necrolysis (TEN) is a rare, life-threatening dermatologic emergency, most commonly triggered by adverse drug reactions. It is characterized by widespread epidermal necrosis, extensive skin sloughing, and frequent mucosal involvement. Here, we present a case of a 73-year-old male with a history of chronic obstructive pulmonary disease (COPD), sarcoidosis, chronic kidney disease, and gout, who developed TEN following ceftriaxone administration for suspected pneumonia. Unlike typical presentations, the patient exhibited widespread epidermal detachment involving 50-60% of total body surface area (TBSA) but lacked mucosal involvement. Diagnosis was confirmed via skin biopsy, which revealed full-thickness epidermal necrosis. Management included the immediate discontinuation of ceftriaxone, supportive care, and wound care strategies, which led to recovery. This case highlights an atypical presentation of TEN, emphasizing that extensive epidermal detachment, even in the absence of mucosal involvement, warrants consideration of TEN as a potential diagnosis. Given the extremely limited reports of ceftriaxone-induced TEN, this case contributes to growing awareness of its potential role in severe cutaneous adverse reactions.

## Introduction

Toxic epidermal necrolysis (TEN) is a rare, severe cutaneous disorder, most often triggered by medications and, less commonly, infections. It is characterized by widespread epidermal detachment and necrosis [1-3]. TEN typically begins with prodromal flu-like symptoms, such as fever, malaise, and sore throat, progressing to painful skin erosions and blisters [2]. Mucosal involvement is present in most cases, often affecting the eyes, mouth, and genitals [2].

The pathogenesis of TEN is well-established and involves primarily CD8^+^ T cell-mediated cytotoxic reactions against keratinocytes, mediated by the granulysin and perforin/granzyme pathways, typically triggered by medications, such as antibiotics, anticonvulsants, and non-steroidal anti-inflammatory drugs (NSAIDs) [1-3]. Genetic susceptibility contributes to risk; for instance, HLA-B5801 and HLA-B1502 alleles are associated with severe cutaneous adverse reactions, though the latter is primarily linked to carbamazepine-induced Stevens-Johnson syndrome (SJS)/TEN in patients of Asian descent [4].

TEN confers a mortality rate of approximately 20-60% [5]. Treatment primarily involves immediate discontinuation of the suspected causative agent and supportive care. Recent evidence supports the use of tumor necrosis factor-alpha (TNF-α) antagonists such as etanercept, which have been shown to reduce mortality and complications [6]. Systemic corticosteroids are typically avoided due to the potential worsening of outcomes. Early recognition and prompt intervention remain critical to improving patient survival.

## Case presentation

A 73-year-old male with a history of chronic obstructive pulmonary disease (COPD), sarcoidosis, chronic kidney disease, and gout was admitted for a COPD exacerbation. In the emergency department, concern for sepsis prompted initiation of vancomycin and cefepime. The following morning, the patient was transitioned to ceftriaxone for suspected community-acquired pneumonia. Approximately 5 h after the first dose, he developed severe pruritus and an erythematous rash on the chest. Ceftriaxone was immediately discontinued, and diphenhydramine was administered for symptomatic relief.

**Figure 1:**
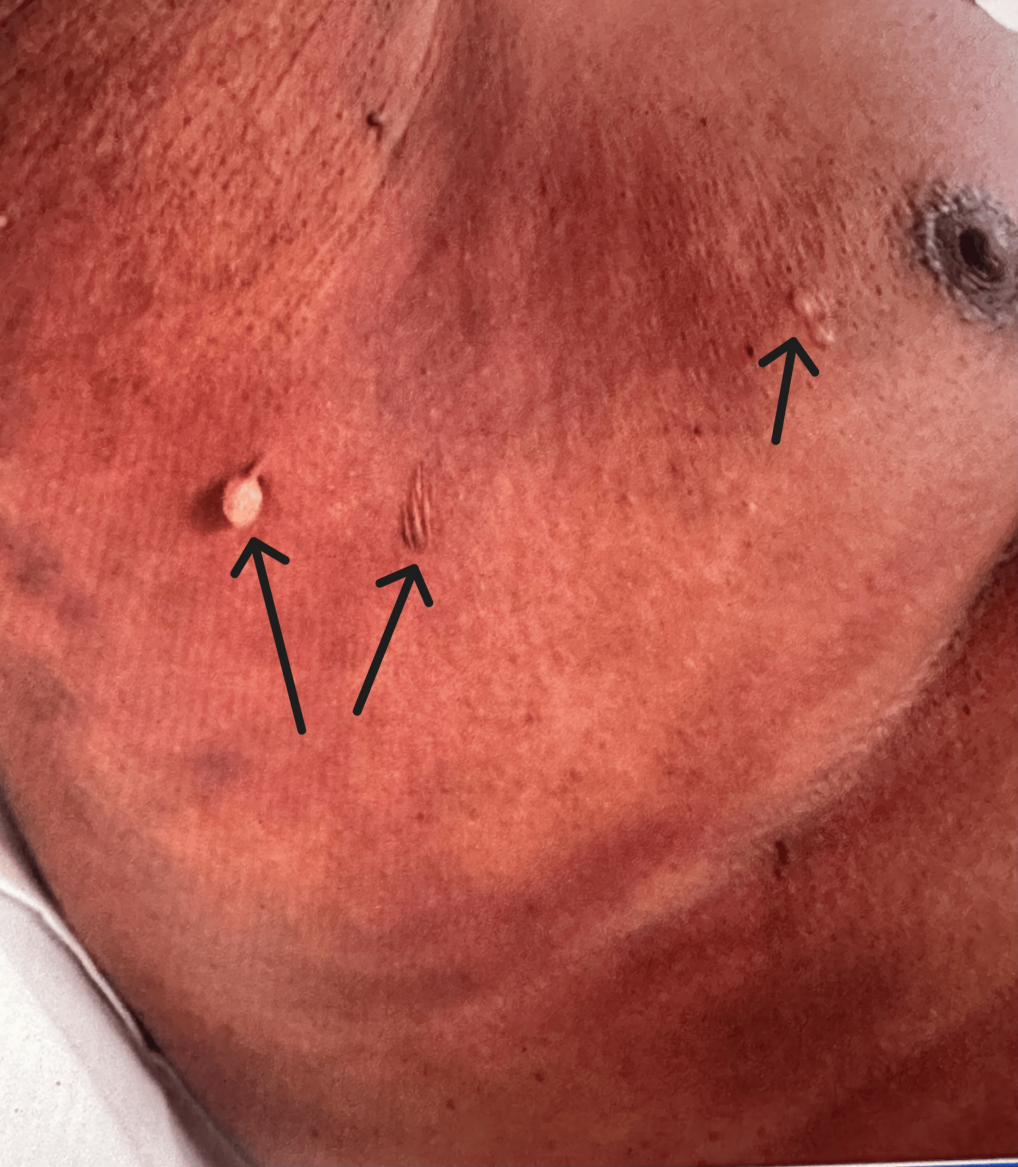
Progression of skin findings from erythematous macules with central dusky discoloration to extensive flaccid bullae (indicated by the arrows).

**Figure 2:**
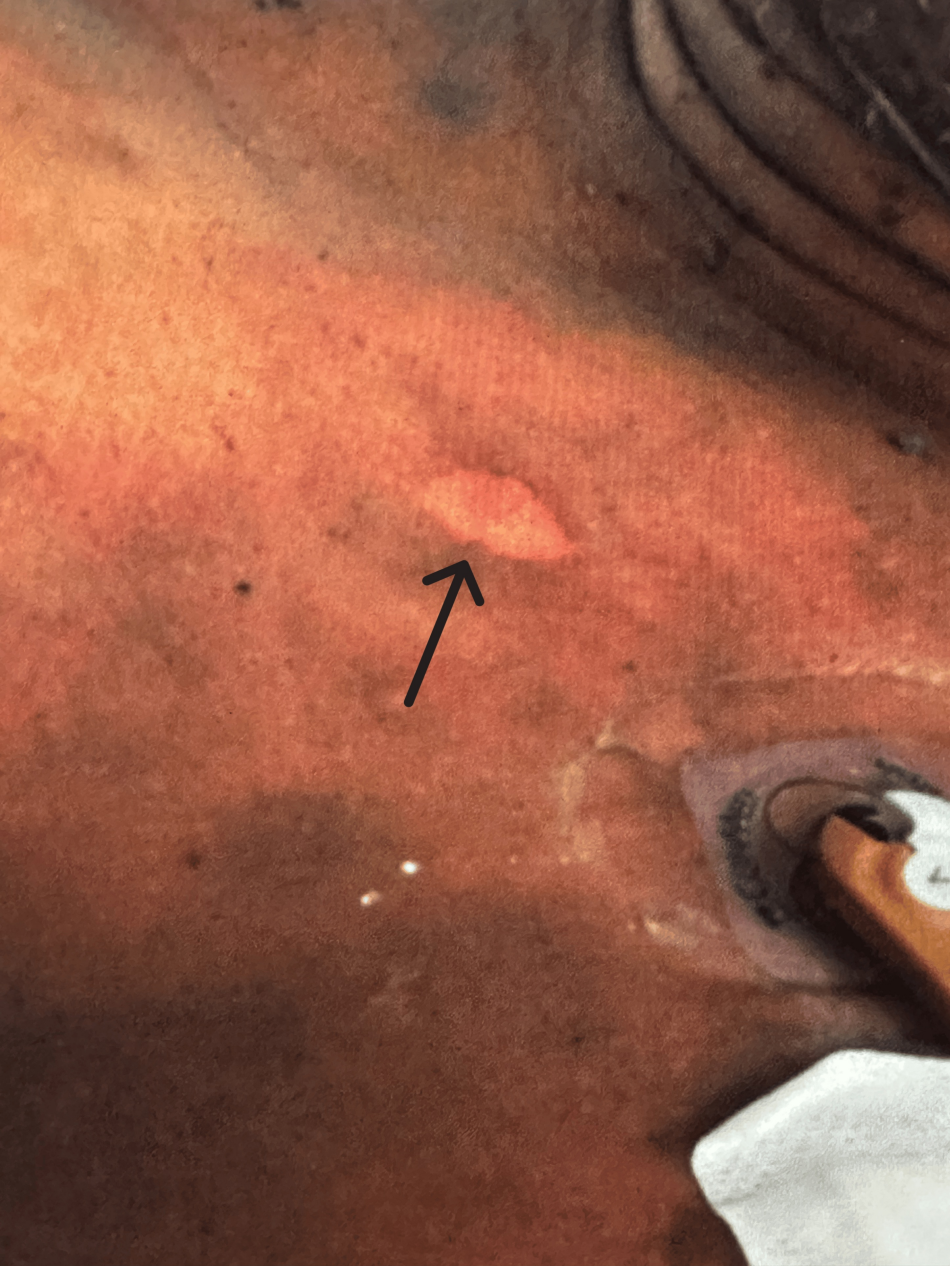
Positive Nikolsky’s sign demonstrated by epidermal shearing and sloughing with gentle lateral pressure, indicative of severe epidermal detachment (indicated by the arrow).

Despite these interventions, the rash progressed over the next three days from erythematous macules with central dusky discoloration to numerous flaccid bullae (Figure 1). Physical examination revealed a positive Nikolsky’s sign and marked tenderness (Figure 2). Skin involvement extended to the lower chest, abdomen, back, hips, and thighs, sparing the arms, face, and mucosal surfaces. Total body surface area (TBSA) involvement was estimated at 50-60% using the “rule of nines” method.

Mucosal evaluation included a detailed assessment by teams from ophthalmology, otolaryngology, and internal medicine. Examination of the oral cavity, eyes, genitals, nasal passages, and pharynx revealed no lesions, confirming mucosal sparing. Dermatology consultation concurred that the temporal relationship between ceftriaxone administration and symptom onset strongly implicated it as the causative agent. A detailed drug timeline is presented below (Table 1).

**Table 1:** Detailed drug timeline for active medications near rash/event onset. NSAIDs: non-steroidal anti-inflammatory drugs

Medication	Timing relative to rash	Outcome
Vancomycin	Started day 0	Continued, no reaction
Cefepime	Started day 0	Stopped day 1, no progression
Ceftriaxone	Started day 1	Rash developed ~5 h after first dose
Diphenhydramine	Given symptomatically	Symptomatic relief only
NSAIDs (none administered)	N/A	N/A

A shave biopsy revealed interface dermatitis with full-thickness epidermal necrosis, confirming a diagnosis on the SJS/TEN spectrum. The algorithm of drug causality for epidermal necrolysis (ALDEN) scoring yielded a score of +5 for ceftriaxone, indicating probable causality. Cefepime received a score of +2 (possible).

Severity-of-Illness Score for Toxic Epidermal Necrolysis (SCORTEN) score on presentation was calculated as 2 (age >40 years, TBSA >30%), indicating a predicted mortality of approximately 12% [5]. Wound care included sterile hydrogel dressings, daily gentle debridement, topical antiseptic ointments, and close monitoring for infection. Supportive care consisted of intravenous fluids, electrolyte management, and temperature regulation. The patient was not treated with corticosteroids.

By hospital day 13, skin lesions demonstrated substantial healing, and the patient was safely discharged. Long-term follow-up at four weeks revealed nearly complete re-epithelialization, accompanied by residual post-inflammatory hyperpigmentation. Human leukocyte antigens (HLA) typing was not performed, and drug lymphocyte stimulation testing (DLST) was deferred due to timing and clinical improvement.

## Discussion

TEN is a rare, life-threatening exfoliative disorder with mortality rates of 20-60% [5]. The most frequent triggers are drug-induced, including NSAIDs, antibiotics, anticonvulsants, and chemotherapeutics [1,4]. Among antibiotics, cephalosporins such as cephalexin, cefuroxime, and ceftazidime are commonly implicated [1]. Ceftriaxone-associated TEN is extremely rare; our systematic literature review included PubMed, Embase, and Cochrane databases from 2000 to 2024 using the search terms “ceftriaxone” AND “toxic epidermal necrolysis” OR “SJS/TEN”. This yielded four previously reported cases, all of which had mucosal involvement [6]. Ceftriaxone-induced TEN without mucosal involvement appears to be exceedingly rare in the research literature.

Differential diagnoses included linear IgA bullous dermatosis, atypical acute generalized exanthematous pustulosis (AGEP), staphylococcal scalded skin syndrome (SSSS), drug reaction with eosinophilia and systemic symptoms (DRESS), and bullous pemphigoid. AGEP was ruled out due to the absence of neutrophilic pustules and rapid progression. Linear IgA disease was excluded based on negative direct immunofluorescence. SSSS was unlikely due to adult age, absence of positive bacterial cultures, and presence of full-thickness epidermal necrosis. DRESS was excluded due to the lack of eosinophilia, systemic involvement, and a latency period. Bullous pemphigoid was excluded due to histopathology findings showing keratinocyte necrosis rather than subepidermal blistering.

Treatment considerations have evolved. Systemic corticosteroids are generally avoided due to evidence suggesting an increased risk of infection and delayed healing [7]. Recent randomized controlled trials demonstrated that etanercept significantly reduced mortality, time to re-epithelialization, and complications in TEN [7]. In our case, given rapid stabilization and TBSA <60%, TNF-α antagonists were not administered.

Mucosal sparing, as observed in our patient, is unusual. Possible mechanisms include differential distribution of drug metabolites, localized immune responses, or variant keratinocyte susceptibility. Clinically, mucosal sparing may signal a less severe disease course and should not exclude TEN from consideration. Recognition of this atypical presentation may refine diagnostic criteria and improve clinical vigilance for drug-induced TEN.

## Conclusions

TEN is a rare, life-threatening cutaneous reaction with high mortality. This case highlights a novel presentation of ceftriaxone-induced TEN without mucosal involvement, emphasizing the need for thorough evaluation of mucosal surfaces and precise causality assessment. Management relies on prompt drug discontinuation, supportive care, and evidence-based interventions such as TNF-α antagonists. SCORTEN scoring provides prognostic insights, and detailed wound care is critical to recovery. Ongoing research is necessary to elucidate pathophysiologic subsets and refine treatment protocols for atypical presentations of TEN.
